# Translations

**Published:** 1880-02

**Authors:** 


### TRANSLATION.

*Progrès Médical.*—(Anonymous.)

At a recent meeting of the Academy of Medicine at Paris, under the presidency of M. Richet, M. Bucquoy read a memoir at once interesting and instructive, bearing upon the accidents of thoracentesis, a subject which has of late been claiming much attention. His paper is entitled, "On pneumothorax without communication of the pleura with the external air, consecutive to thoracentesis by aspiration." In two cases of which M. Bucquoy reports the observations, he recognized the signs of pneumothorax after thoracentesis, without any maladroit manœuvre (presumably on the part of the operator), a defect of the apparatus or a pulmonary perforation having given access to the air into the plueral cavity. The first time it was in a pleurisy purulent and of very long standing, the patient being tuberculous; the pneumothorax developed itself after the second puncture. The operation for empyema showed that gases were mingled with the purulent liquid, and the autopsy that there was no perforation. The second fact is a case of acute pleurisy, in which, in the middle of the operation, a sharp, whistling sound revealed suddenly that gas was rushing into the apparatus. There was consecutive pneumothorax, but the pleurisy healed none the less rapidly. M. Bucquoy explained the development of the pneumothorax under these conditions by the setting at liberty of gases dissolved in the liquid of effusion, under the influence of the aspiration and of the vacuum thus established in the cavity of the pleura itself. This phenomenon necessarily supposes that the retreat of the lung continues after the evacuation of a part of the serous fluid.

Two similar cases are reported by MM. Lereboullet and Tennesson; they trace to the same cause. This accident, rare, but little known, and consecutive to thoracentesis, deserves to be noted, for when it happens during the course of an operation it frightens singularly the physician, who might otherwise think that air was entering the pleura. But, as to consecutive pneumothorax thus produced, it is an accident of sufficient minor gravity that the possibility of this complication should not be a contra-indication for thoracentesis in chronic pleurisy.

M. Burg then read from a work having for title: "Metallotherapy at Vichy, from 1870 to 1875, in diabetes and subsidiarily of its association to alkaline medication, in order to augment and correct its effects." According to M. Burg, metallotherapy is destined to be of undoubted value in the treatment of diabetes, and probably also of several other affections of a diabetic character where an alkaline base of treatment is justifiable. Iron and arsenic play, in alkaline waters which contain them, a role disregarded until to-day, and lessened at the expense of alkaline medication. It is to these two metals, inconsiderately administered, or without any other reason than that of habit or fashion, and consequently to metallotherapy, that certain patients are indebted for not having experienced the inconveniences of alkaline medication. M. Burg terminates his communication by the following conclusions: "A nervous affection, diabetic, for example, being given, the whole treatment consists in applying a means, whatever it may be, but a metal by preference, when it can be done, which, be it *intus* or *extra*, can bring back the sensibility and muscular force to their normal condition."

---

In the Society of Biology, M. Fr. Franck presented the result of his experiments upon the reflex respiratory influence of the sensible nerves of the heart. The author, after having laid bare and nude the section of the depressor nerve of a rabbit, excited the central end of the nerve. He saw produced at once an extreme amplitude of respiratory movements; there was a permanent augmentation of thoracic aspiration. M. Frank made still another interesting experiment. In the right heart of an animal he made to arrive through the jugular vein a gum sound containing a solution of chloral. As soon as the liquid fell into the auricule, the respiration sustained an immediate arrest.

---

In the Society of Surgery, M. Ledentu presented a patient upon whom he carried out with success resection of the knee for a fungous phlegmasia alba dolens. About five centimetres of the femur were taken off, and two centimetres of the tibia; the patella was removed. The limb was placed rigorously in a solid plaster apparatus with Lister's dressings; no sutures of the bone were applied; the cure became complete; however, the osseous alterations were such that, during the operation, M. Ledentu was in doubt as to amputation not being preferable.

